# Sum of phase-shifted sinusoids stimulation prolongs paralyzed muscle output

**DOI:** 10.1186/s12984-020-00679-1

**Published:** 2020-04-10

**Authors:** Kristen Gelenitis, Max Freeberg, Ronald Triolo

**Affiliations:** 1 Department of Biomedical Engineering, Case Western Reserve University, 10,900 Euclid Avenue, Cleveland, OH 44106 USA; 2 Case Western Reserve University School of Medicine, Cleveland, OH USA; 3 Department of Orthopaedics, Case Western Reserve University, Cleveland, OH USA; 4 Advanced Platform Technology Center, Louis Stokes Cleveland Veterans Affairs Medical Center, Cleveland, OH USA

**Keywords:** Neuroprostheses, Paralysis, Stimulation, Fatigue, Duty cycle, Feedback control

## Abstract

Neuroprostheses that activate musculature of the lower extremities can enable standing and movement after paralysis. Current systems are functionally limited by rapid muscle fatigue induced by conventional, non-varying stimulus waveforms. Previous work has shown that sum of phase-shifted sinusoids (SOPS) stimulation, which selectively modulates activation of individual motor unit pools (MUPs) to lower the duty cycle of each while maintaining a high net muscle output, improves joint moment maintenance but introduces greater instability over conventional stimulation. In this case study, implementation of SOPS stimulation with a real-time feedback controller successfully decreased joint moment instability and further prolonged joint moment output with increased stimulation efficiency over open-loop approaches in one participant with spinal cord injury. These findings demonstrate the potential for closed-loop SOPS to improve functionality of neuroprosthetic systems.

## Introduction

Paralysis due to spinal cord injury (SCI), stroke, or other upper motor neuron injury often leads to limited mobility and wheelchair dependence. The resulting sedentary lifestyle has numerous negative health consequences including muscle deconditioning, decreased bone density, poor circulation, pressure injuries, and cardiovascular disease [1]. Weight-bearing standing exercise can address these health concerns, allow those that are wheelchair dependent to obtain level eyesight with their peers, and expand their sphere of interaction with the world.

Standing after upper motor neuron injury can be achieved with neural stimulation that activates the paralyzed musculature of the lower extremities [2, 3]. However, due to the rapid muscle fatigue commonly induced by extracellular stimulation [4, 5] and exacerbated by physiological changes after paralysis [6], standing times with current systems are limited and highly variable. In our laboratory, the median standing time for participants with SCI is three minutes [3], and similarly short durations have been reported by others despite various interventions for improvement [7–10]. These systems are therefore mainly beneficial for short duration tasks, such as transfers into and out of a wheelchair. Extending standing times would enable users of neuroprostheses to obtain greater physiological, functional, and social benefits from these systems.

**Fig. 1:**
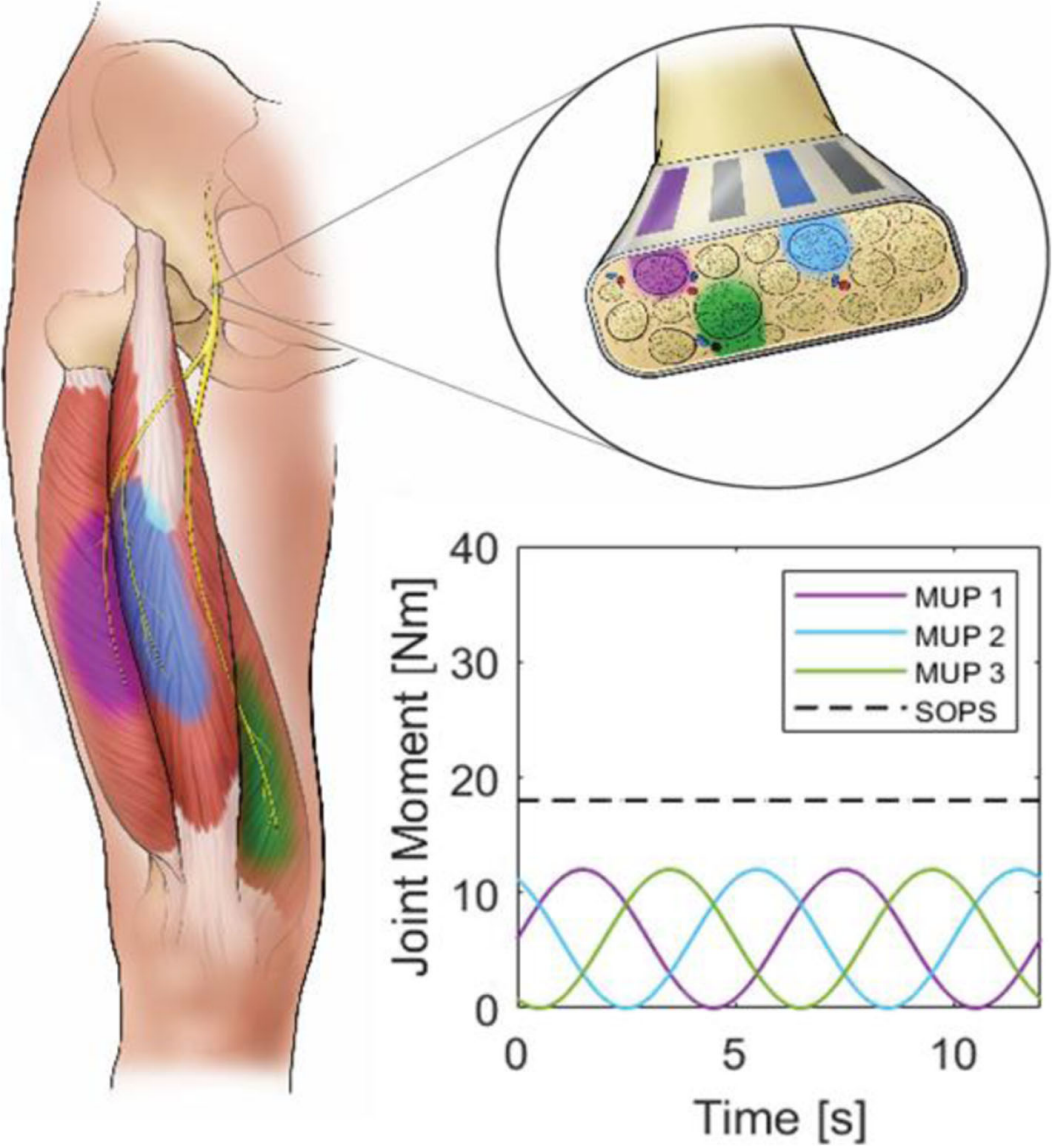
Sum of Phase-shifted Sinusoids stimulation. Three independent MUPs are activated by independent electrode contacts to produce sinusoidal moments individually. When activated together, the independent, phase-shifted sinusoids sum to a constant moment output greater than the peak moment from any one MUP individually

Conventional approaches deliver non-varying stimulus current waveforms through multiple knee extensor-activating electrode contacts to ensure the joint remains locked and stable during standing. However, simultaneously activating many knee extensor fibers continuously contributes to the rapid muscle fatigue and short standing times [11, 12]. Previous work shows implanted extra-neural cuff electrodes with multiple independent contacts can selectively activate independent yet synergistic populations of motor units [2, 13–15]. This selectivity enables a novel technique for prolonging paralyzed muscle output and extending standing durations, termed Sum of Phase-shifted Sinusoid (SOPS) stimulation [16]. SOPS stimulation modulates the pulse width (PW) delivered through each contact such that each independently activated motor unit pool (MUP) produces a sinusoidal joint moment. When these PW patterns are phase-shifted from each other and delivered through multiple contacts simultaneously, the resulting total joint moment is constant with a value greater than the peak moment from any MUP individually (Fig. 1). SOPS stimulation therefore reduces the duty cycle of each MUP by allowing brief periods of rest while maintaining a higher overall net moment.

SOPS stimulation showed moderate improvements in sustaining joint moment but significantly higher knee moment instability compared with constant stimulation in a prior study [16]. Instability arises from dynamic changes in the muscle over time that cause the exact stimulation patterns necessary for perfect sinusoidal summation to vary. To improve the stability of joint moment output, this study introduces a closed-loop SOPS control scheme and compares it directly with open-loop SOPS and conventional constant stimulation in one subject with SCI previously implanted with a selective nerve-based stimulation system. We hypothesized that closed-loop SOPS stimulation would prolong joint moment compared to open-loop SOPS or constant stimulation and increase stability over open-loop SOPS.

## Methods

### Participant

One 25-year-old male participant with C7 AIS-C SCI received an implanted stimulator in his abdomen and bilateral 8-contact composite flat interface nerve electrodes (C-FINEs) around the proximal femoral nerves of both legs 2 years prior to this study. Three contacts per C-FINE, found to selectively activate independent knee extensor MUPs in a previous study [14], were used in the stimulation patterns investigated. All tests were approved by the local Institutional Review Board.

### Experimental design

While seated on a robotic dynamometer (Biodex, Shirley NY), the investigated limb’s knee joint center was aligned with the center of a six degree-of-freedom load cell (JR3 Inc., Woodland, CA) and fixed at 20 degrees of flexion from the horizontal. Single leg knee extension moment produced from C-FINE stimulation at 20 Hz was recorded and normalized by body weight (60 kg). Pulse amplitude (PA) was 0.8 mA for each contact and test condition. Custom Simulink models on a MATLAB real-time xPC Target adjusted stimulus PW between 0 and 255 μs based on test condition. Conventional stimulation tests used non-varying trains of PWs that produced one-third of the target moment on individual MUPs, such that when activated together initial total moment would approximate target moment.

Sinusoidal moments *y(t)* produced by stimulation through individual contacts in SOPS schemes followed the equation:

$$ y(t)=\frac{M}{3}\sin \left(\frac{2\pi }{T}t+\varphi \right)+\frac{M}{3} $$

where the target moment M = 18 Nm, period T = 6 s, and phase shift *φ* = 0, $\frac{2\pi }{3}$, and $\frac{4\pi }{3}$, for the first, second, and third contact respectively. This produced phase-shifted sinusoidal moments that, when combined, would approximate the constant target moment value (Fig. 1).

**Fig. 2:**
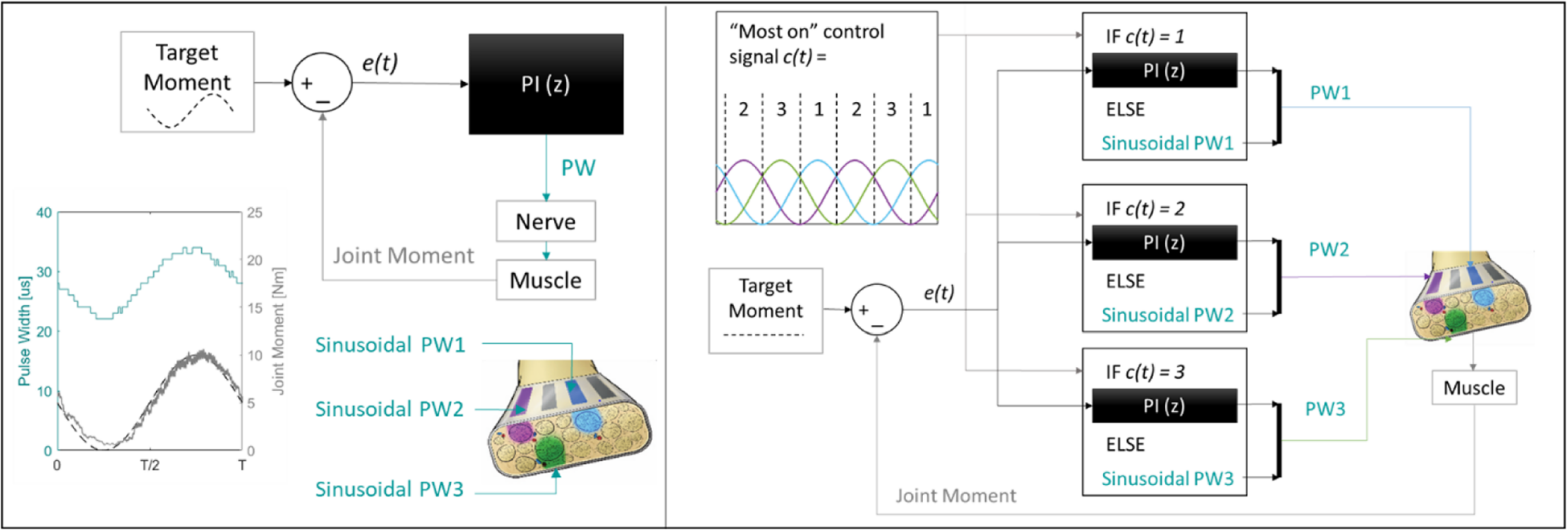
(LEFT) Open-loop SOPS stimulation scheme. Pulse width vectors that produce sinusoidal moments through individual contacts were determined by tracking the desired sinusoid with a PI controller. PW vectors that successfully tracked the sinusoid with low RMS error for each contact were saved and implemented continuously during open-loop SOPS. (RIGHT) Closed-loop SOPS control scheme. Individually tuned PI controllers adjust stimulation to the “most on” MUP at each time point to maintain a constant target moment. Other MUPs continue to receive PW vectors that produce sinusoidal moments on that contact

In open-loop SOPS stimulation, a PW pattern that induced the desired sinusoidal knee moment on each individual contact was determined by tracking that sinusoid with a PI controller (Fig. 2). PI gains were manually tuned for each contact using an organized heuristic procedure. With the proportional (*P*) and integral (*I*) gains initially set to zero, *P* was increased until the controller PW output induced knee moments that closely tracked the frequency and amplitude of the desired sinusoid with low oscillatory noise. A *P* value that resulted in less than one Newton-meter (Nm) absolute difference between actual and desired sinusoid peaks and oscillations less than 1 Nm peak-to-peak was considered acceptable. Once a suitable *P* value was found, *I* was adjusted such that noise and overshoot further decreased but delays in sinusoid tracking greater than 0.2 s (T/30) at the peaks were avoided. Tuned PI controller PW outputs that resulted in the lowest RMS error between actual knee moment and the desired sinusoidal moment were saved for each contact. These PW vectors were then delivered through each contact simultaneously such that the individual sinusoids were offset from each other and the estimated resulting moment would be near-constant at the target value (Fig. 2). These PW vectors were repeated throughout the fatigue trial without change in the open-loop SOPS condition.

In closed-loop SOPS stimulation, the same PW vectors used in open-loop SOPS were initially delivered through each contact. Total joint moment feedback from the load cell was monitored within the Simulink stimulation model (Fig. 2). An error signal *e(t)* quantified the difference between actual and target knee moment. PI controllers within the closed-loop model, tuned using the same gains determined for each contact prior to open-loop implementation, adjusted the PW value sent to the “most on” contact to minimize *e(t)*. Only the PW through the “most on” contact was adjusted at each time point because it is assumed that the MUP activated by that contact is contributing the most to the overall knee moment at that particular instant, and is thus likely the MUP that is fatiguing. By increasing PW to maintain overall joint moment throughout the trial, closed-loop SOPS stimulation can recruit new fibers as the originally recruited fibers fatigue, and produce a total joint moment that matches the target moment with increased stability and duration.

Constant, open-loop SOPS, and closed-loop SOPS stimulation trials were performed on both legs of the participant (*n* = 2) over three consecutive days of testing. Constant testing was performed on the first day, followed by open-loop and closed-loop SOPS so that baseline performance with conventional stimulation could be assessed prior to any cumulative fatigue to prevent bias. Each fatigue trial lasted for at least 10 min. Knee moment and PW output data was analyzed using custom MATLAB code. Time to fatigue (TF) and time below target (TBT) were determined as the time knee moment fell below 0.135 Nm/kg, the approximate knee moment required for standing [17], and the target moment of 0.3 Nm/kg respectively. Target moment was chosen as 0.3 Nm/kg to ensure all stimulation conditions would remain above the minimal knee moment required for standing for a measurable period of time even as they fatigue. The 0.3 Nm/kg target could be produced independently by each individual MUP in this participant, so it was considered an appropriate compromise between functional requirements for standing (0.135 Nm/kg), and what was consistently attainable without fully recruiting the muscle to ensure a dynamic range for controller PW modulation in closed-loop trials. Total work (W) is defined in this study as the knee moment integrated over time. Though there is no net movement, energy expenditure and work done by a muscle is proportional to the force-time integral [18, 19], which is proportional to the moment-time integral in a fixed, isometric contraction. Cumulative charge (Q) injected and stimulation cost (C), the ratio of charge injection to work performed (Q/W), were also assessed. Moment ripple (R) was calculated as the ratio of the range in moment to the average moment over each consecutive six-second window, to encompass a full sinusoidal period in the SOPS conditions. The R for each six-second window within the 10-min trials from both legs [*n* = 200] for each stimulation condition [m = 3] comprised the nxm matrix for statistical testing. R data was found to be homogenous (Brown-Forsythe *p* > 0.05), but non-normal (Chi-squared *p* < 0.05). A Kruskal-Wallis test was thus used for statistical analysis of R, followed by post-hoc Dunn’s tests with MATLAB’s *multcompare* function.

## Results

**Fig. 3:**
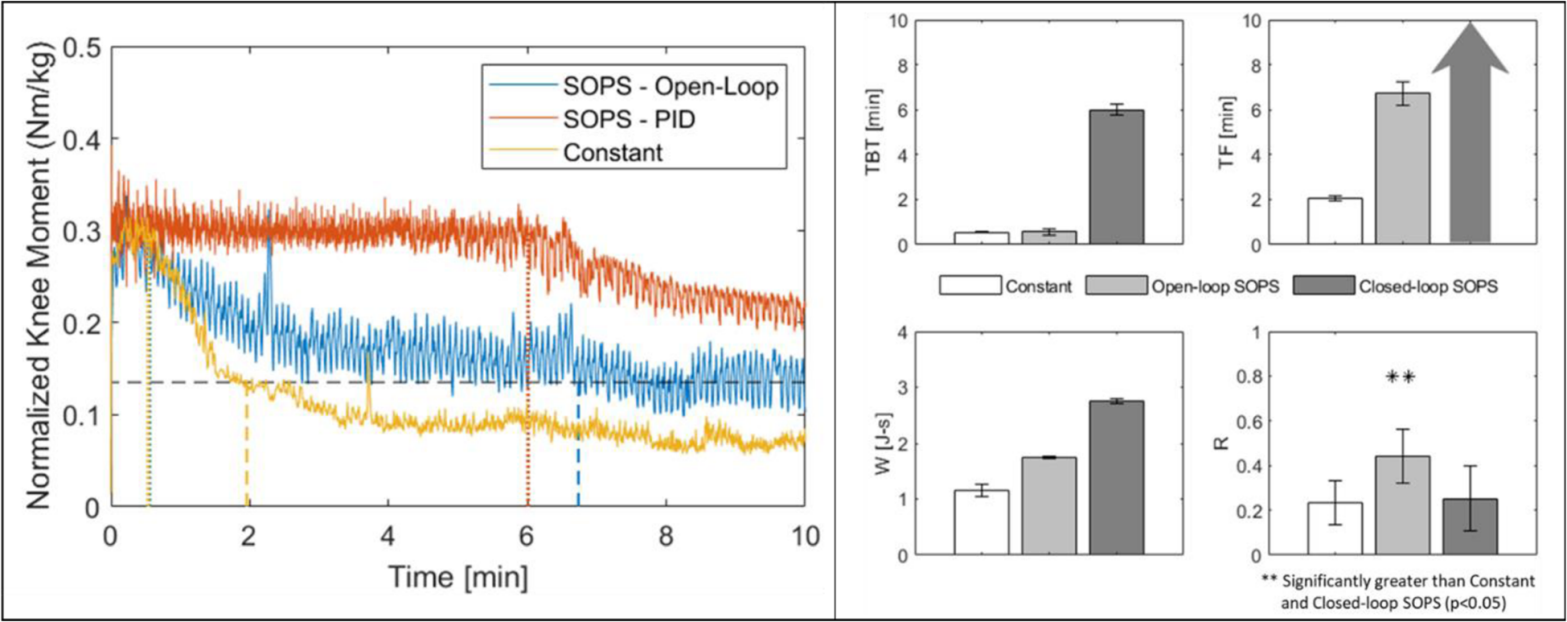
(LEFT) Average left and right leg moment output over time from each stimulation condition, normalized to participant body weight. Dashed vertical lines indicate average time to fatigue (TF) below 0.135 Nm/kg (dashed horizontal line). Dotted vertical lines indicate average time below target (TBT) knee moment, 0.3 Nm/kg. (RIGHT) Comparison of outcome measures from constant, open-loop SOPS, and closed-loop SOPS stimulation trials. Bar height indicates left and right leg mean. Error bars indicate standard deviation. Closed-loop SOPS increases time below target (TBT, upper left). Both SOPS paradigms increase total work (W, bottom left) and time to fatigue (TF, upper right) over constant stimulation. Arrow indicates closed-loop SOPS did not fall below TF value by the end of the trial for either leg. Ripple index (R, bottom right) was significantly higher with open-loop SOPS compared to the other conditions. No significant difference in ripple was found between constant and closed-loop SOPS

Left and right knee moment averages over time for each stimulation condition show both open and closed-loop SOPS stimulation prolonged TF and increased W over constant stimulation (Fig. 3). Additionally, closed-loop SOPS stimulation prolonged TBT over the other conditions and decreased R with statistical significance compared with open-loop SOPS. In fact, closed-loop control decreased the R prevalent in open-loop SOPS to the point of no statistically significant difference with conventional constant stimulation.

**Fig. 4:**
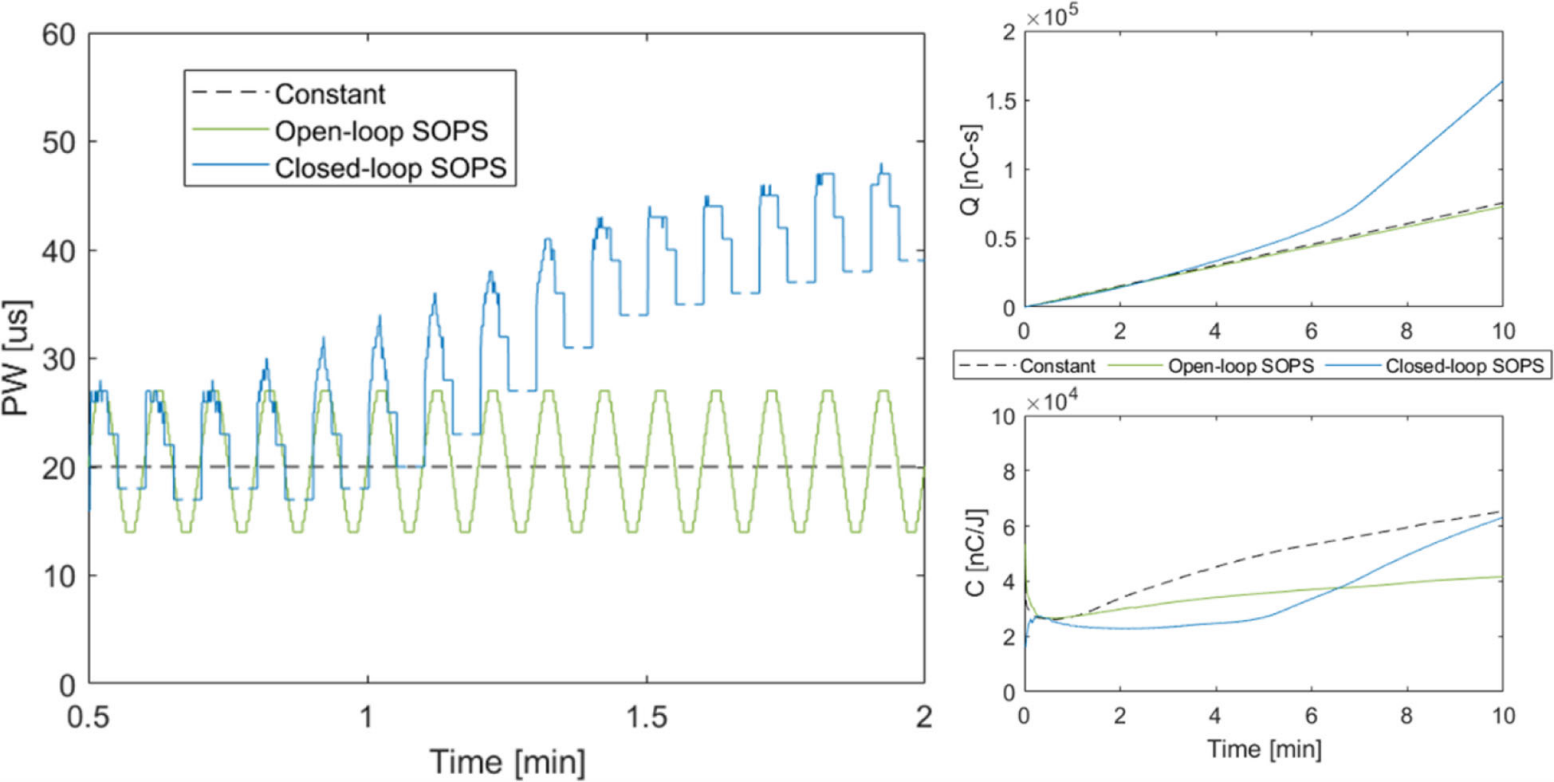
(LEFT) PW vectors delivered through the same contact for each stimulation condition between 0.5 and 2 min, where the moment produced from each paradigm diverge. As the MUP fatigues and moment decreases, closed-loop SOPS increases PW to maintain overall target moment. (TOP RIGHT) Left and right leg average Q with each stimulation condition. Q initially accumulates linearly and consistently across all conditions, but sharply increases with closed-loop SOPS in the last half of the trials. (BOTTOM RIGHT) C, the ratio of Q to W throughout the trial. Closed-loop SOPS maintains lower cost despite higher cumulative charge due to higher comparative moment output. Both SOPS stimulation paradigms are more efficient than constant stimulation

Initially, each condition delivered equivalent charge through the C-FINE (Fig. 4). After the first minute, the closed-loop SOPS scheme began to increase PWs through certain contacts as needed, resulting in higher Q than the other conditions in the second half of the trials. However, both open- and closed-loop SOPS maintained a lower C than constant stimulation.

## Discussion

SOPS stimulation reduced duty cycle by enabling one MUP to rest or minimally contribute while other MUPs maintained the desired joint moment. Duty cycle is approximately reduced by the inverse of the number of contacts included in the paradigm. In this study, contribution from three independent yet synergistic MUPs reduced the duty cycle of each by 33%. Reducing duty cycle with SOPS increased TF and W over constant stimulation (Fig. 3) in our participant, in accordance with several other studies [20–22] that reported decreased duty cycles prolong muscle output and delay stimulation-induced fatigue. Duty cycle reduction and rotating activation among separate MUPs is thought to delay glycogen store depletion and metabolite build-up, and create a pumping action to promote blood flow and oxygen delivery to the muscle [23], which may explain the improvements in joint moment maintenance with the SOPS paradigm.

Despite clear improvements in TF and W, open-loop SOPS significantly increased R over constant stimulation, as was found in prior investigation [16]. This would likely be unacceptable during standing, as participants may perceive large fluctuations in knee moment as unstable even if they remain above the threshold for locked knee extension. Closed-loop SOPS implementation successfully reduced moment ripple to the point of no significant difference with constant stimulation (Fig. 3) by adjusting PW to match a stable target moment value. Additionally, closed-loop SOPS stimulation progressively increased PWs as MUPs began to fatigue (Fig. 4) to recruit non-fatigued fibers and further prolong target moment output. Though this results in more charge being injected throughout the trial, closed-loop SOPS still remains a more efficient paradigm in terms of charge injected per unit of work performed. Lower stimulation cost may improve the efficiency and battery life of portable or implantable neuroprostheses.

The dynamometer provided feedback for closed-loop stimulation in this seated isometric study. For SOPS stimulation to be successful in functional tasks, such as standing, walking, or exercising, portable sensors capable of determining either overall joint moment or individual contributions from each MUP must be identified. Wearable electromyography (EMG), accelerometers or inertial measurement units (IMU), and mechanomyography (MMG) sensors have shown promise in other movement control applications [24, 25] and may be incorporated into closed-loop SOPS paradigms in future studies.

## Conclusions

SOPS stimulation prolongs maintenance of joint moment, increases work performed by muscles prior to fatigue, and increases stimulation efficiency compared to constant stimulation. Closed-loop control of the SOPS paradigm provides significant improvements in joint stability and further prolongs moment output by recruiting additional MUPs as needed. This study is limited as just two legs of a single participant were investigated during seated isometric leg extension. However, results indicate clear improvements in studied outcome measures, and merit further investigation of SOPS techniques with additional participants and for other applications. This study also highlights the advantages of real-time feedback for the control of neuroprostheses and furthers the demand for reliable wearable sensing technology.

## Data Availability

The datasets used and/or analyzed in the current study are available from the corresponding author upon reasonable request. Direct correspondence to kxg277@case.edu

## Abbreviations

SOPS: Sum of Phase-shifted Sinusoids
MUP: Motor unit pool
SCI: Spinal cord injury
PW: Pulse width
C-FINE: Composite flat interface nerve electrode
PA: Pulse amplitude
TF: Time to fatigue [min]
TBT: Time below target [min]
W: Total work [J]
R: Moment ripple index
Q: Cumulative charge [nC-s]
C: Stimulation cost [nC/J]
EMG: Electromyography
IMU: Inertial measurement unit
MMG: Mechanomyography
